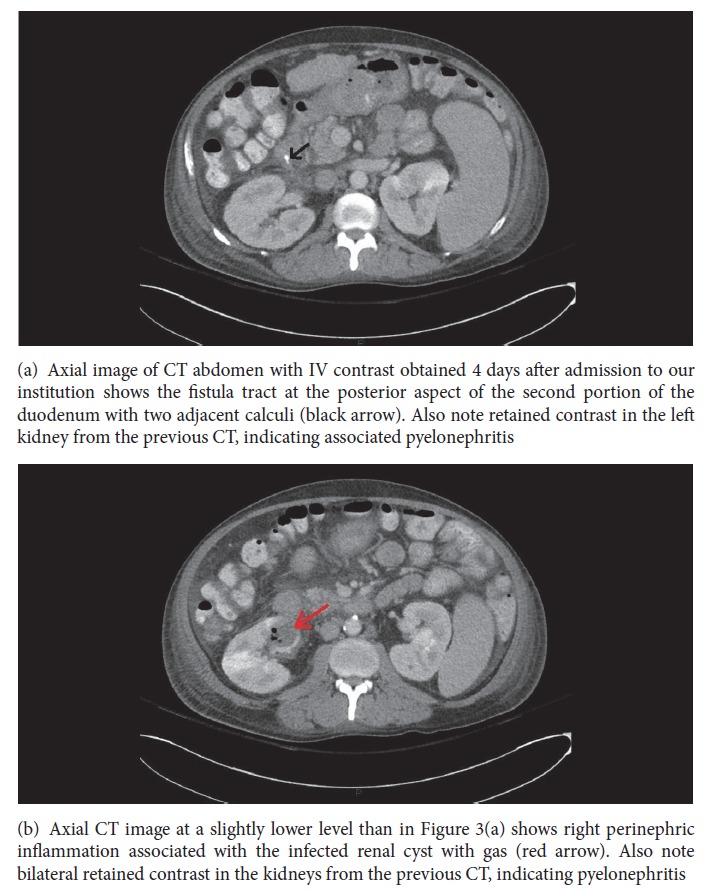# Infected Renal Cyst as a Complication of Dropped Gallstones during Laparoscopic Cholecystectomy

**DOI:** 10.1155/2018/2478245

**Published:** 2018-09-30

**Authors:** Chelsea Kennedy-Snodgrass, Vivian Keenan, Douglas S. Katz

**Affiliations:** ^1^NYU Winthrop Hospital, Department of Internal Medicine, 259 1st Ave, Mineola, NY 11501, USA; ^2^NYU Winthrop Hospital, Department of Pulmonary and Critical Care, 259 1st Ave, Mineola, NY 11501, USA; ^3^NYU Winthrop Hospital, Department of Radiology, 259 1st Ave, Mineola, NY 11501, USA

## Abstract

Dropped gallstones are a relatively common complication, occurring in 3% to 32% of laparoscopic cholecystectomies performed, depending on various intraoperative risk factors. However, complications arising from dropped gallstones are relatively rare, occurring in fewer than 1% of such patients, and can include abscesses and inflammatory masses confined to the subhepatic space, presenting days to years later. We report a patient who developed an infected renal cyst as a result of dropped gallstones, which created a fistula from the duodenum to a previously simple right renal cyst, which was initially identified on an abdominal CT scan. Dropped gallstones can result in substantial morbidity in a minority of patients following cholecystectomy performed for cholecystitis, and a high clinical as well as radiological index of suspicion may be required for accurate early recognition and treatment.

## 1. Introduction

One of the most common major abdominal surgical procedures performed is laparoscopic cholecystectomy (LC), which is the standard of care for routine gallbladder removal [1, 2]. Overall, complication rates for LC are lower than for open cholecystectomy, likely reflecting that open procedures are reserved for complex elective cases or for complicated laparoscopic procedures which require conversion to an open cholecystectomy [3, 4]. LC, however, more frequently results in injury to the common bile duct and complications from dropped gallstones [3]. Complications which can arise from dropped gallstones in the setting of cholecystitis include abscesses and inflammatory masses which are generally confined to the subhepatic space or the retroperitoneum below the subhepatic space; however, uncommon and unusual complications have also occurred including broncholithiasis, hernia sac stones, and calculi migrating near or into the ovaries or fallopian tubes [3, 5, 6]. We describe an unusual case of a patient who developed an infected renal cyst as a result of dropped gallstones, which created a fistula from the duodenum to a previously simple right renal cyst, which was identified on an abdominal computed tomography (CT) performed 59 days following laparoscopic cholecystectomy.

## 2. Case Presentation

A 45-year-old male presented to our hospital with diaphoresis, chills, and worsening right flank pain. He had a past medical history of cirrhosis due to alcoholism and portal hypertension with esophageal varices managed with prior transjugular intrahepatic portosystemic shunt (TIPS) procedure. He had a history of hepatic encephalopathy, hypertension, and type II diabetes. He had a recent admission to another institution for septic shock secondary to recurrent cholecystitis and had recently had an LC fifty-eight days prior. The patient was admitted to the outside hospital fifty-nine days prior to admission to our institution for subacute cholecystitis, which had initially required a cholecystostomy drain on prior admissions, and then ultimately an LC at that hospital. The gallbladder was not noted to be perforated, but the procedure was complicated by dropped gallstones, some of which were retrieved as stated in the operative report. On the current admission, the patient was hypotensive requiring vasopressors and was anemic and thrombocytopenic, requiring blood and platelet transfusions.

**Figure 1:**
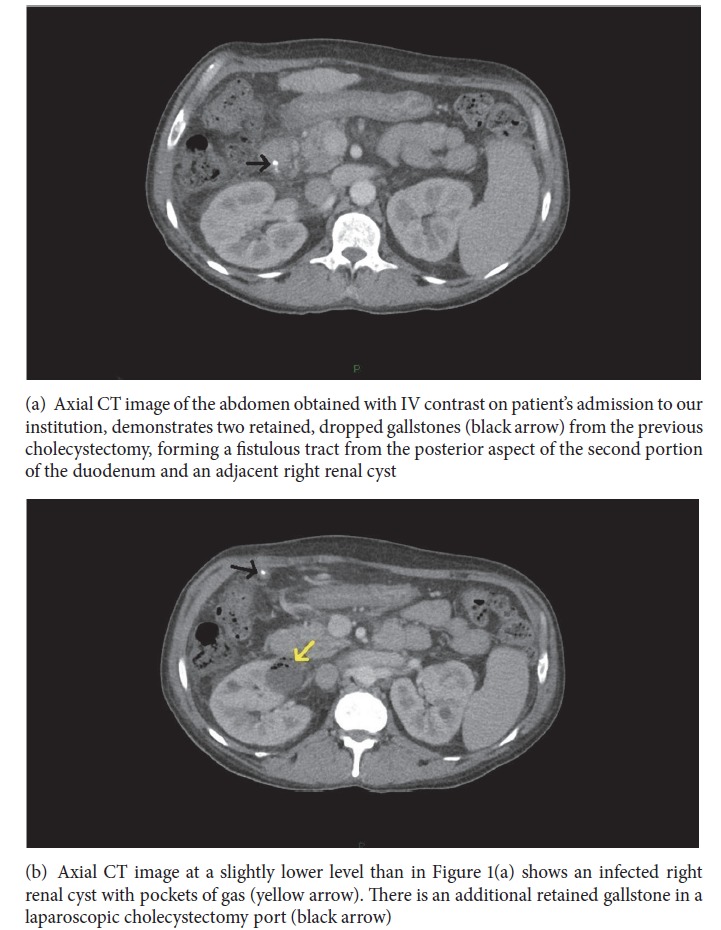


**Figure 2:**
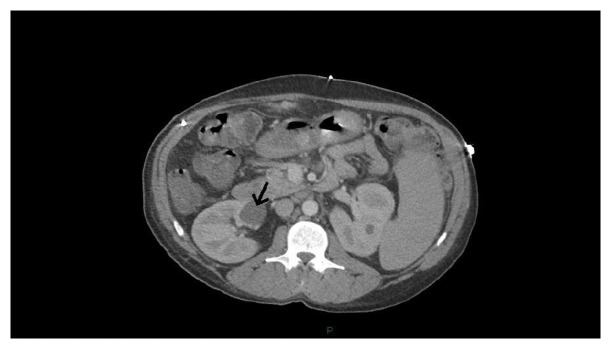
Axial image from CT of the abdomen with IV contrast obtained 65 days prior to admission revealed a simple right renal cyst (black arrow) in the location of the now infected cyst.

A CT scan of the abdomen and pelvis with IV contrast was performed on admission demonstrating residual gallstones in the gallbladder fossa and/or cystic duct remnant and multiple small fluid collections and/or forming granulomatous masses surrounding additional retained/dropped gallstones in the surgical tracts and vicinity. A fistula with gallstones was also seen extending through the posterior and inferior wall of the second portion of the duodenum, to the anterior and superior aspect of a right renal cyst, which measured 3.4 cm x 3.0 cm and which contained gas in its superior aspect (Figures 1(a) and 1(b)). A previous CT of the abdomen and pelvis performed with IV contrast sixty-five days prior to admission and seven days prior to the LC demonstrated a simple right renal cyst in the location of the now complex and infected cyst, measuring 2.8 cm x 2.5 cm (Figure 2).

Drainage of the infected renal cyst seen on the initial CT was considered; however, the cyst was relatively small and inaccessible. A repeat CT of the abdomen and pelvis with oral and IV contrast was performed four days after admission due to concern for abscess formation as the patient continued to have right flank pain. The CT demonstrated an unchanged superinfected cyst in the right kidney, with the fistula tract still visible, and heterogeneous retention of IV contrast in the right kidney, which was consistent with associated pyelonephritis (Figures 3(a) and 3(b)).

On initial presentation, the patient was noted to have a history of* Klebsiella pneumoniae* and vancomycin-resistant enterococcus (VRE) in the cholecystectomy drain and was treated for the gallstone abscess and fistula accordingly with meropenem as there was no other source of infection. Blood cultures were later positive for* K. pneumoniae* and VRE. The patient was ultimately treated with linezolid and meropenem was deescalated to ceftriaxone.

Discussion between the patient's outside hepatologist, the abdominal radiologist, the interventional radiologist, and the gastroenterologists determined that the most likely etiology of the initial sepsis was an infected renal cyst secondary to an infected dropped gallstone. The patient was ultimately transferred to the outside hospital where he previously had his cholecystectomy for surgical follow-up.

## 3. Discussion

Laparoscopic cholecystectomy is the current accepted standard of treatment for symptomatic gallbladder disease [7]. Although morbidity rates for laparoscopic cholecystectomy are lower than for open cholecystectomy, complications can include bile duct injury, biliary leakage, and infection due to dropped gallstones in the setting of acute or chronic cholecystitis [7–9]. It is thought that gallbladder perforation and subsequent dropped gallstones may occur more often during LC than during open cholecystectomy due to excessive traction on the gallbladder during dissection from the liver, accidental entrance into the gallbladder during the dissection itself, or the elevated pressures on the gallbladder as it is extracted from the abdomen through a small incision [2, 10]. The incidence of gallbladder perforation reported varies widely, from 3% to 32%; however, the incidence of clinically significant events due to dropped gallstones is rare, occurring in fewer than 1% of such patients [1, 2, 8, 10–12].

Dropped gallstones are often located near the liver, in Morison's pouch, in the gallbladder fossa, and in the pelvis, but occasionally can be found elsewhere, including the left abdomen and even the thorax, due to concurrent fistulization [1]. Such dropped gallbladder calculi in the setting of cholecystitis can be retrieved intraoperatively by irrigating and suctioning the abdominal/pelvic cavity, by collection with a sponge, or recovered postoperatively by percutaneous interventional radiologic, laparoscopic, or open surgical techniques [7]. Recovery can be difficult as was demonstrated in our patient, as the operative report noted that all dropped gallstones were thought to have been retrieved. Dropped gallstones have a low incidence of complications, especially in the setting of a routine cholecystectomy not performed for acute or subacute infection, but can potentially cause a range of postoperative complications [3]. In the setting of acute cholecystitis, dropped gallstones are more likely to have infectious complications [13]. The most common complication of dropped gallstones is intraperitoneal abscess formation with or without transabdominal fistula [11]. These abscesses or inflammatory masses containing the gallstones or gallstone fragments are generally limited to the subhepatic space or to the retroperitoneum below the subhepatic space [5]. Uncommon presentations of spilled gallstones have included urinary tract involvement, obstructive cholangitis, secondary acute appendicitis, and even intestinal volvulus or intestinal obstruction [11]. These complications from dropped calculi may not present until many years following the initial surgery in some patients [3]. Several cases have been reported of abscesses occurring a decade after LC with gallstone spillage due to Actinomyces infection [14–16]. Due to the wide range of possible complications and the potentially extended time-interval between cholecystectomy and clinical presentation, dropped gallstones may not be considered in the differential diagnosis, leading to inaccurate diagnosis and inappropriate treatment.

In patients with suspected spilled gallstones, ultrasound and especially CT are recommended for evaluation of possible abscesses and associated complications [5]. Ultrasound may be a more sensitive modality for detection of dropped gallstones; however, imaging depth is limited due to beam penetration [17]. Gallstones with high calcium content will appear as high attenuation foci on CT; however, pure cholesterol stones may go undetected [1]. Unenhanced CTs should be used for stone identification as adjacent inflammation may enhance with contrast and obscure the stone [17]. When diagnosing complications resulting from dropped gallstones, it is important that their characteristic CT appearance be recognized, because such calculi with associated collections or granuloma formation can mimic other diseases, including tumors or bowel obstruction [8].

Our patient is the first reported case, to our knowledge, of an infected renal cyst resulting from dropped gallstones in the setting of cholecystitis, with a fistula between the duodenum and a previously simple renal cyst. This case highlights the diverse range of complications and therefore the wide variety of presenting signs and symptoms which can occur from spilled gallstones in this setting. Though rare as a major complication, dropped gallstones during laparoscopic cholecystectomy can result in significant morbidity, and a high clinical as well as radiological index of suspicion may be required for early recognition and treatment.

## Figures and Tables

**Figure 3:**